# An anoikis-related gene signature for prediction of the prognosis in prostate cancer

**DOI:** 10.3389/fonc.2023.1169425

**Published:** 2023-08-17

**Authors:** Xiaodong Zhao, Zuheng Wang, Zilu Tang, Jun Hu, Yulin Zhou, Jingping Ge, Jie Dong, Song Xu

**Affiliations:** ^1^ Jinling School of Clinical Medicine, Nanjing Medical University, Nanjing, Jiangsu, China; ^2^ Department of Urology, Eastern Theater General Hospital of Medical School Of Nan Jing University, Nanjing, Jiangsu, China

**Keywords:** anoikis-related gene, signature, prostate cancer, biochemical recurrence, prediction

## Abstract

**Purpose:**

This study presents a novel approach to predict postoperative biochemical recurrence (BCR) in prostate cancer (PCa) patients which involves constructing a signature based on anoikis-related genes (ARGs).

**Methods:**

In this study, we utilised data from TCGA-PARD and GEO databases to identify specific ARGs in prostate cancer. We established a signature of these ARGs using Cox regression analysis and evaluated their clinical predictive efficacy and immune-related status through various methods such as Kaplan-Meier survival analysis, subject work characteristics analysis, and CIBERSORT method. Our findings suggest that these ARGs may have potential as biomarkers for prostate cancer prognosis and treatment. To investigate the biological pathways of genes associated with anoikis, we utilised GSVA, GO, and KEGG. The expression of ARGs was confirmed by the HPA database. Furthermore, we conducted PPI analysis to identify the core network of ARGs in PCa.

**Results:**

Based on analysis of the TCGA database, a set of eight ARGs were identified as prognostic signature genes for prostate cancer. The reliability and validity of this signature were well verified in both the TCGA and GEO codifications. Using this signature, patients were classified into two groups based on their risk for developing BCR. There was a significant difference in BCR-free time between the high and low risk groups (P < 0.05).This signature serves as a dependable and unbiased prognostic factor for predicting biochemical recurrence (BCR) in prostate cancer (PCa) patients. It outperforms clinicopathological characteristics in terms of accuracy and reliability. PLK1 may play a potential regulatory role as a core gene in the development of prostate cancer.

**Conclusion:**

This signature suggests the potential role of ARGs in the development and progression of PCa and can effectively predict the risk of BCR in PCa patients after surgery. It also provides a basis for further research into the mechanism of ARGs in PCa and for the clinical management of patients with PCa.

## 1 Introduction

Prostate cancer (PCa) is the most common malignancy in men, and the rate of occurrence is significantly associated with age (1). In recent years, the number of new cases and the number of deaths from PCa have remained high, and the age of the population affected has been getting progressively younger (2). For patients with early-stage disease, surgery can often achieve good results (3), while for patients with late-stage disease, almost every treatment plan is associated with serious side effects that can only benefit a small number of people (4). Despite the availability of many therapies, PCa remains incurable. In the treatment process, biochemical recurrence (BCR) is often an unavoidable clinical phase. In PCa and other tumours, active surveillance is an effective intervention. While proper intervention can significantly delay disease progression, overtreatment can cause serious complications. Achieving a balance between avoiding the adverse effects of overtreatment and achieving early disease detection, and trading minimal quality of life for maximum survival benefit, is an unavoidable problem.

The detection of the prostate specific antigen (PSA) is the most commonly used index for the active surveillance of patients with PCa. BCR was defined as a rise in PSA level of 0.2 ng/ml or more in patients after prostatectomy (RP) with an increasing trend on two consecutive tests or an increase of more than 2 ng/ml from the PSA nadir in patients treated with RT, and all patients were free of clinically and/or radiologically detectable lesions (5). Statistically, BCR occurs in 30-50% of PCa patients treated with RT (6) and in 20-40% of patients treated with radical RP (7). BCR is a significant risk factor for PCa distant metastases, specificity and overall mortality (8). Approximately 30% of patients with BCR will develop clinically manifest distant metastases, resulting in 19% to 27% of patients dying within 10 years (9). In addition, it may be useful in guiding the design of appropriate treatment and follow-up strategies for patients at higher risk. Although other indicators such as Gleason score and PSA may predict prognosis in PCa patients, their ability to predict BCR risk is limited. Previous studies have shown that the AUC and C-index of PSA for predicting the risk of biochemical recurrence in PCa patients are both less than 0.75, and the AUC of Gleason score is only 0.715 (10, 11).Therefore, the identification and construction of a more accurate and specific risk signature is of great importance to guide the diagnosis, treatment and follow-up of PCa patients.

**Figure 1:**
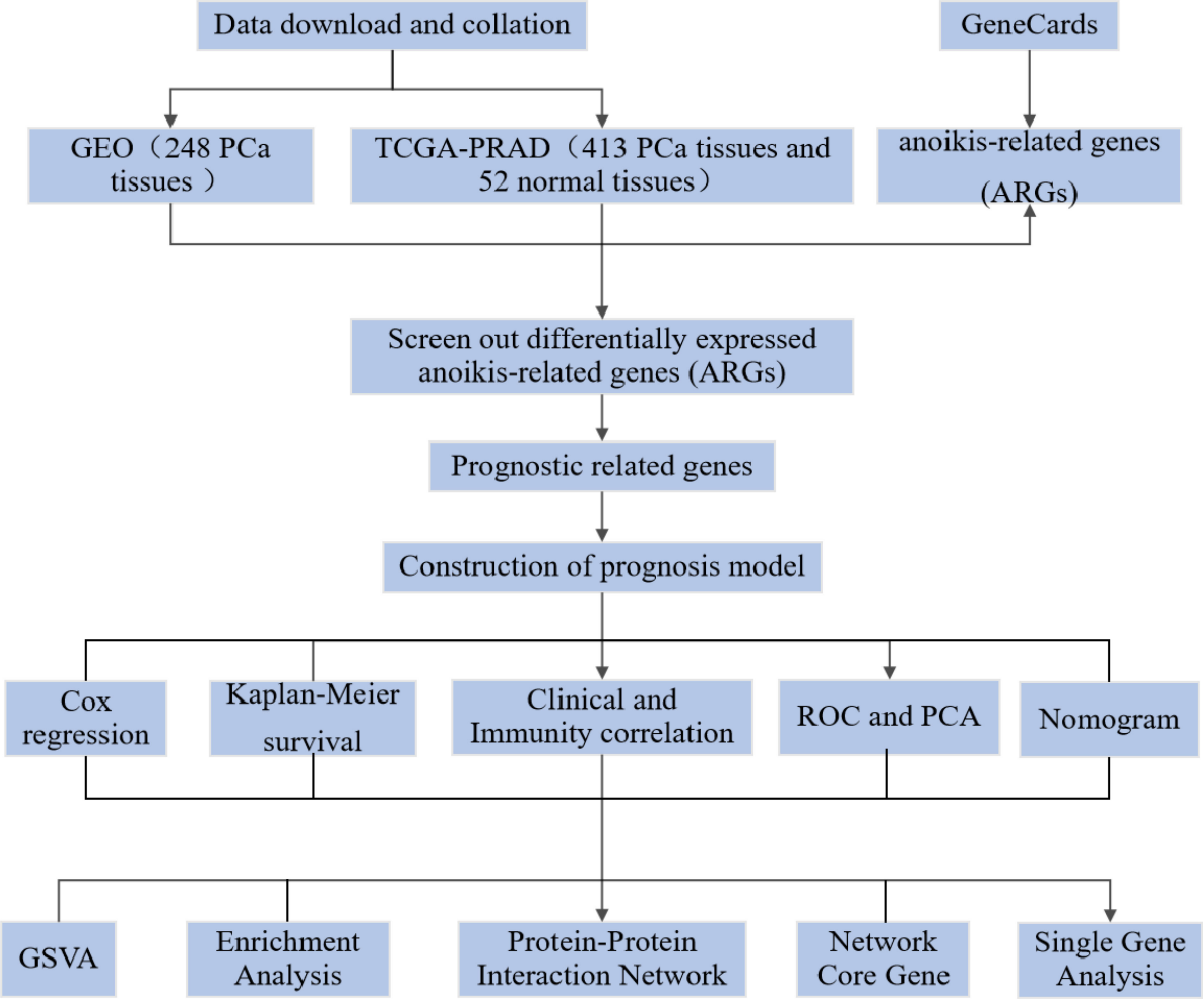
The flow diagram of this study.

In the onset and development of PCa, genes play an important regulatory role. Differences in the prognosis of PCa patients result from the interaction of genetics and environment (4). In monitoring the status of PCa and evaluating the response to treatment, the detection of gene expression levels has great potential. To assess patient risk and provide scientific guidance for targeted treatment, many genes have been included in guidelines for gene testing. For example, autophagic and scorch-related gene expression levels were used to try to predict BCR risk in PCa patients (9, 12). Anoikis is an apoptotic process induced by loss of cell adhesion to the extracellular matrix and is another form of programmed cell death. Establishing anoikis resistance is a key link to tumour interstitial transformation, which can significantly promote tumour initiation and progression (13). In normal prostate tissue, by inducing programmed apoptosis of epithelial cells separated from the extracellular matrix, anoikis can preserve the normal tissue structure of the prostate. The occurrence and development of PCa may be promoted by the vascularisation of the extracellular matrix and the production of anoikis resistance (14). There is no relevant research on the use of ARGs to predict the prognosis of PCa, although some studies have shown that anoikis is related to the prognosis of several common tumours. Our aim is to investigate a possible association between ARG expression and prognosis in PCa patients (**Figure 1**).

## 2 Methods

### 2.1 Data preparation

The data in this study were derived from two datasets. The training set was derived from the TCGA database and included transcriptomic and clinical data of 413 PCa patients with BCR information. The validation set, including data from 248 PCa patients, was derived from the Gene Expression Omnibus (GEO) database (GSE116918). The search term “anoikis” was used to extract ARGs from the GeneCard database, and genes with a correlation score of 1.00 have been excluded (**Supplementary Table 1**). Clinical and pathologic information from both cohorts is shown in **Table 1**. R (version 4.2.1) was used for analysis.

**Table 1:** Clinical characteristics of two cohorts of patients with PCa.

Variables	TCGA dataset (n=413)	GEO dataset (n=248)
Age (%)		
≤65	297 (71.91)	87 (35.08)
>65	116 (28.09)	161 (64.92)
cT stage (%)		
T1-2	298 (72.16)	127 (51.21)
T3-4	44 (10.65)	96 (38.71)
Unknow	71 (17.19)	25 (10.08)
cM stage (%)		
M0	389 (94.19)	NA
M1	2 (0.48)	NA
Unknow	22 (5.33)	NA
pT stage (%)		
T1-2	151 (36.56)	NA
T3-4	257 (62.23)	NA
Unknow	5 (1.21)	NA
pN stage (%)		
N0	293 (70.94)	NA
N1	68 (16.46)	NA
Unknow	52 (12.60)	NA
Residual tumor (%)		
No	261 (63.20)	NA
Yes	130 (31.48)	NA
Unknow	22 (5.32)	NA
Gleason score (%)		
≤7	241 (58.35)	107 (43.15)
>7	172 (41.65)	141 (56.85)
BCR status		
BCR	48 (11.6)	56 (22.58)
BCR-free	365 (88.4)	192 (77.42)

TCGA, The Cancer Genome Atlas; GEO, Gene Expression Omnibus; cT, clinical tumor; cM, clinical metastasis; pT, pathology tumor; pN, pathology node; BCR, biochemical recurrence; NA, Not Available.

### 2.2 Identification of differentially expressed genes

Heatmap comparison of expression levels of anoikis-related genes in PCa and normal prostate tissues. The DEGs of the ARGs between the PCa tissues and the normal prostate tissues were evaluated using the limma software in R (fold change (FC) > 1.5 together with the false discovery rate (FDR) < 0.05).

### 2.3 Prognostic anoikis-related signature construction and validation

Prognostic ARS was constructed from TCGA database and validated in TCGA and GEO databases. First, candidate prognostic genes were obtained from DEGs by univariate Cox regression analysis. Prognostically related genes were displayed in a forest map (p<0.05). In order to minimise the risk of over-fitting, the prognostic signature was established by means of Lasso-Cox regression analysis and then verified by means of Lasso penalty analysis. The list of signature genes (**Table 2**) and the formula for the calculation of the risk score were then exported. Furthermore, the ARS signature genes were preliminarily examined and verified at the protein level using the Human Protein Atlas (HPA) site (https://www.proteinatlas.org/).

**Table 2:** Eight ARGs greatly correlated with BCR-free survival outcome.

Gene	Coefficient	Hazard ratio (95% CI)	P value
EGF	-0.3185	0.545 (0.391−0.759)	<0.001
MYC	0.0053	1.436 (1.025−2.011)	0.035
PLK1	0.2349	2.501 (1.768−3.540)	<0.001
EZH2	0.1989	2.745 (1.790−4.211)	<0.001
AFP	0.2917	2.100 (1.324−3.330)	0.002
NOX4	0.0870	2.020 (1.443−2.826)	<0.001
BMP6	0.0483	1.662 (1.301−2.124)	<0.001
MMP11	0.2756	1.743 (1.429−2.125)	<0.001


risk score=sum(expression of the ARGn×coefficient)

Risk scores were calculated for each sample based on signature genetics risk factors, and patients were divided into low and high risk groups based on median risk scores. The R package “pheatmap” was used to plot risk curves and survival status maps to show the BCR in the high and low risk groups. PCA analysis was performed using “ggplot2” R package to evaluate distribution of gene expression levels in each group in signature. “Survival” and “survminer” were used for survival and PFS analysis. In order to test the effectiveness of the signature, the K-M survival curve and the time-ROC curve were plotted using the R package “timeROC”. Univariate and multivariate analyses were also performed to evaluate the predictive power of the signature. Finally, we explored whether there were differences in patients’ risk scores between different immunophenotypes or clinical characteristics using the “ggpubr” R package for clinical correlation analysis and immunophenotyping analysis.

### 2.4 Nomogram construction and verification

To further predict the efficacy of BCR for PCa patients, we attempted to construct a nomogram using the R package “rms” by considering the risk score and clinicopathological characteristics. The predictive validity and clinical applicability of the nomogram were evaluated by calibration curve analysis and survival analysis. Finally, an independent prognostic analysis was performed to determine whether the nomogram could be used as an independent prognostic indicator.

### 2.5 Gene set variation analysis and gene set enrichment analysis

In order to observe the expression and enrichment of the pathways in the high and low risk groups, we performed gene set variation analysis using the R package “GSVA”. Subsequently, the gene ontology (GO) function enrichment analysis and the Kyoto encyclopedia of genes and genomes (KEGG) pathway enrichment analysis were carried out.

### 2.6 Interacting protein network and network core genes

A protein interaction network was constructed by analysing protein interactions between ARGs using the STRING database. We then performed single gene analysis and clinical correlation analysis using ROC curves for each candidate core gene.

### 2.7 Statistical analyses

Statistical analysis was performed using R software version 4.0.2 and various R software packages. Mean ± standard deviation and percentage were used for continuous variables and categorical variables with normal distribution, respectively. Significant statistical difference was defined as P<0.05 (two-tailed).

## 3 Result

### 3.1 To screen for candidate genes related to prognosis

**Figure 2:**
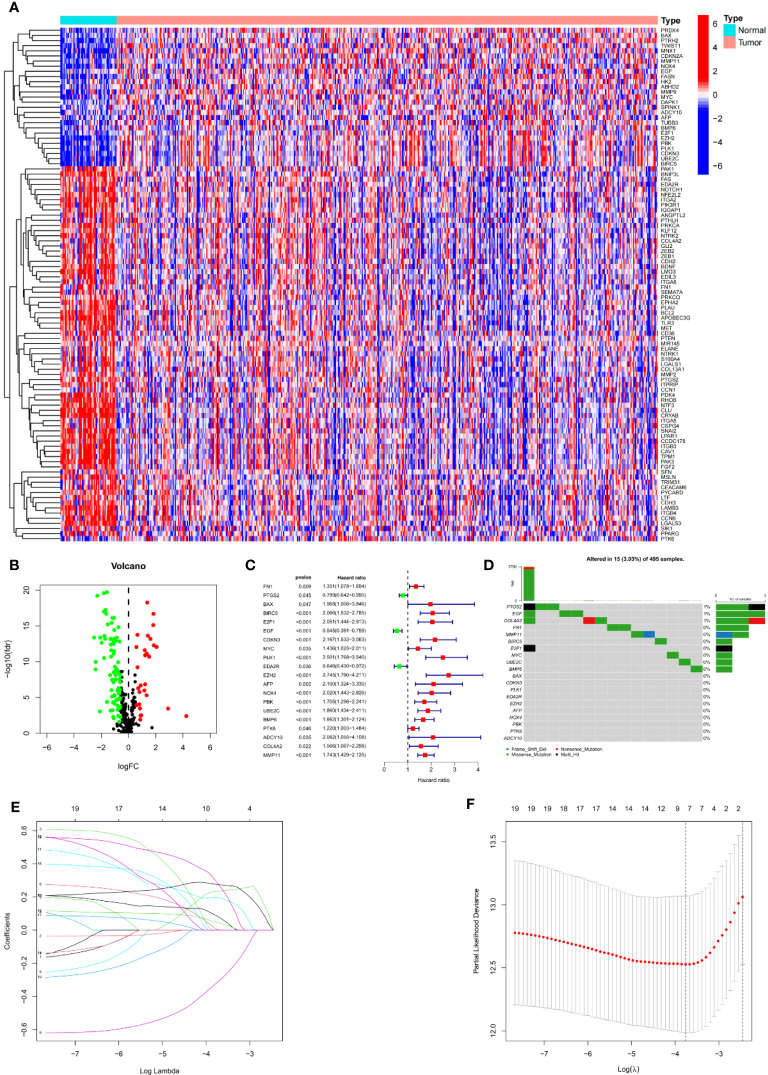
Establishment of prognostic features in PCa. **(A)** Heatmap depicts the variations ARGs expression. **(B)** Volcano plot of differentially expressed genes (DEGs) in PCa. **(C)** Forest map shows prognosis related genes. **(D)** Forest map shows prognosis related genes. **(E)** LASSO coefficient of ARGs in PCa. **(F)** LASSO regression analysis for the development of ARS.

We first calculated the expression level of 337 ARGs extracted from the gene map of 413 tumuor tissues and 52 normal tissues, and then searched for the corresponding differentially expressed genes. 27 genes were found to be upregulated and 73 genes downregulated by PCa. A heat map depicted the differentially expressed DEGs (**Figure 2A**). Volcano plot (**Figure 2B**) shows the differentially expressed genes (DEGs).

### 3.2 Prognostic signature construction and validation

**Figure 3:**
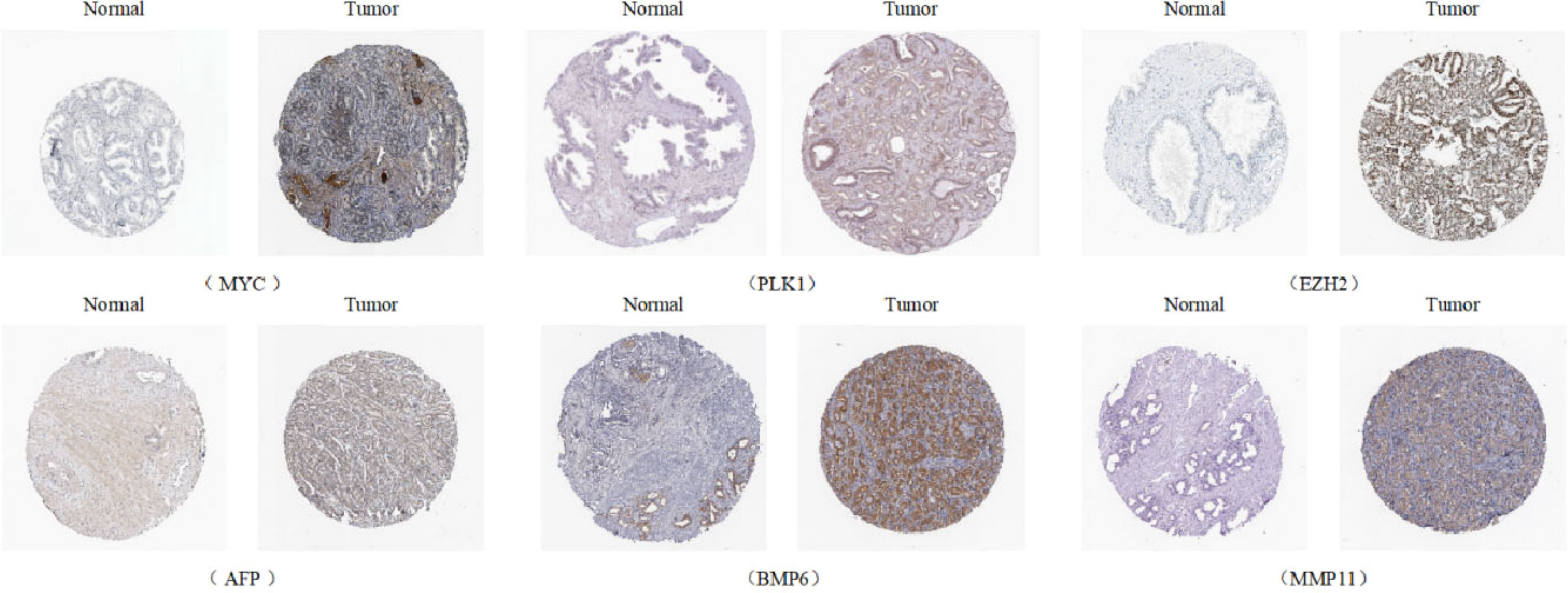
Immunohistochemical results of the genes of ARS (Antibody Staining) (Human Protein Atls Database, https://www.proteinatlas.org/).

**Figure 4:**
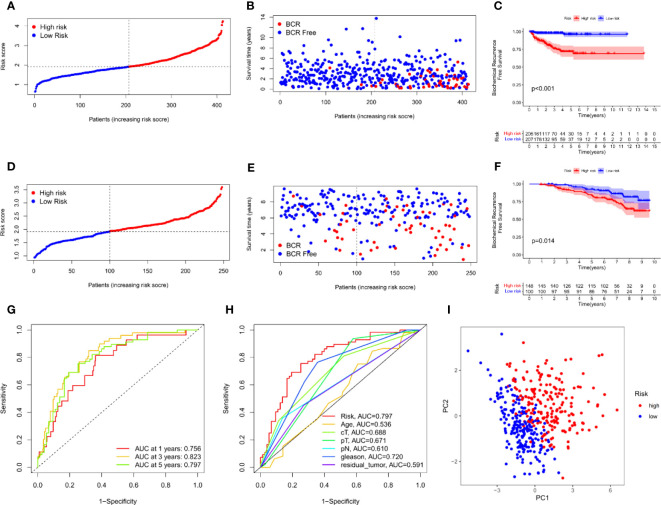
Prognostic accuracy of the ARS. **(A–C)** The evaluation of prognostic characteristics in TCGA cohort: **(A)** The layout of increasing risk scores. **(B)** The curve of BCR status. **(C)** Kaplan–Meier curves of survival outcome between two groups. **(D–F)** The evaluation of prognostic characteristics in GEO cohort: **(D)** The layout of increasing risk scores. **(E)** The curve of BCR status. **(F)** Kaplan–Meier curves of survival outcome between two groups. **(G)** ROC curves of predictive performance of the ARS in TCGA cohort. **(H)** ROC curve of prognosis signature and clinical factors. **(I)** The two risk groups were distinguished by principal component analysis (PCA) in TCGA cohort.

**Figure 5:**
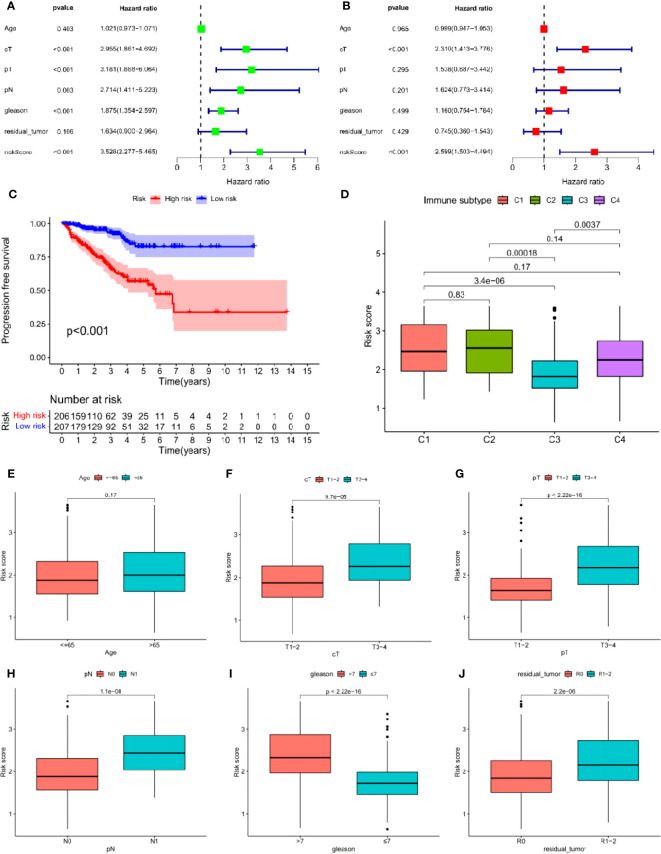
**(A, B)** Cox regression analysis: **(A)** Univariate cox regression analysis. **(B)** Multivariate cox regression analysis. **(C)** Progress free survival analysis. **(D)** Analysis of immunological classification. **(E–J)** The relationship between the signature and different clinical features: **(E)** Age. **(F)** Clinical tumor. **(G)** Pathology tumor. **(H)** Pathology node. **(I)** Gleason. **(J)** Residual tumor.

Univariate Cox analysis was used to obtain 20 prognosis-related ARGs (**Figures 2C, D**).To assess collinearity of these 20 genes, Lasso Cox regression analysis was used to determine prognostic signature of 8 ARGs (EGF, MYC, PLK1, EZH2, AFP, NOX4, BMP6, MMP11) (**Figures 2E, F**, **Table 2**). Immunohistochemistry results of 8 signature genes were obtained from the HPA database (**Figure 3**): MYC, PLK1, EZH2, AFP, BMP6 and MMP11 in prostate and PCa tissues. PLK1 was highly expressed in PCa tissues and MYC, EZH2, AFP and MMP11 were more highly expressed in PCa tissues, which was basically consistent with the signature of the prognostic risk score. However, due to differences in detection methods and lower sample size, BMP6 expression was slightly lower in PCa. ARS prediction performance was verified in both testing (TCGA) and validation (GEO) sets. According to the prognostic risk score signature, the risk scores of patients from the test and verification sets were calculated. The samples in each cohort were divided into two high-risk and low-risk groups in accordance with the median value of the risk score (**Figures 4A, B**). Consistent with the expected results of the signature, the number of patients with BCR increased as the risk score increased (**Figures 4D, E**). The analysis of BCR-free survival (**Figures 4C, F**) showed that the BCR-free survival rate of the patients gradually decreased over time, and there were significant differences in the BCR-free survival rate of the high-risk and low-risk groups in the two cohorts (P<0.01). The AUC for BCR-free survival at one year, three years and five years were 0.756, 0.823 and 0.797, respectively, showing an excellent predictive performance (**Figure 4G**). The AUC of the risk score was 0.797, significantly outperforming clinicopathological features, although univariate Cox analysis showed that clinical T stage, pathological T stage, N stage and Gleason score all had the potential to predict patient prognosis (**Figures 4H**, **5A**). Principal component analysis (PCA) showed that the signature gene could effectively discriminate the patients in the high-risk group from the low-risk group (**Figure 4I**). A further multivariate analysis showed that the signature gene could be used as an independent prognostic indicator for the patients with PCa (**Figure 5B**). The results of the progression-free survival (PFS) analysis are shown in **Figure 5C**. Immunotype analysis showed that the risk score of patients in the C3 group was much lower than in the other groups (P<0.05), with no statistical difference in the risk score between the other groups (**Figure 5D**). **Figures 5E–J** show the outcome of the clinical correlation analysis. Apart from age, there were significant differences in risk scores at different stages of other clinical characteristics.

### 3.3 Nomogram construction and validation

**Figure 6:**
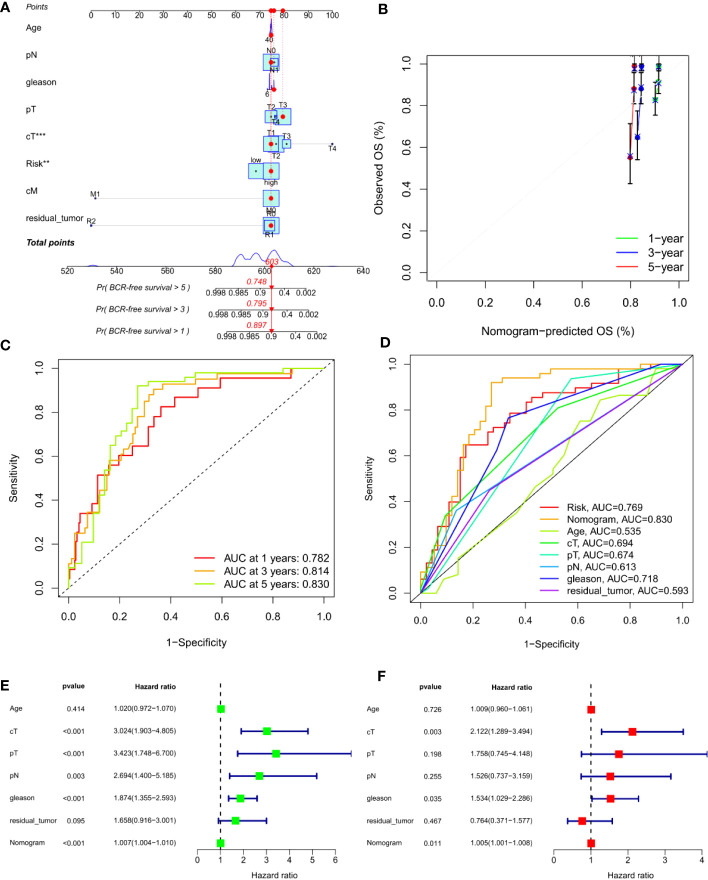
Construction of an ARS-based nomogram. **(A)** A nomogram for predicting BCR-free survival in the TCGA cohort. **(B)** Nomogram calibration plots for predicting OS at 1, 3, and 5 year in the TCGA cohort. **(C)** Time-dependent ROC analysis curve for the nomogram. **(D)** ROC curve of nomogram, prognosis signature and clinical factors. **(E)** Univariate Cox analysis. **(F)** Multivariate Cox analysis. **, P<0.01, ***, P<0.001.

To predict the 1-, 3- and 5-year BCR incidence in PCa patients, we constructed a nomogram based on risk scores and clinicopathological features (**Figures 6A, C**). Areas under the curve (AUCs) remained significantly above 0.75 (**Figure 6D**). The calibration curves demonstrated the consistency between the nomogram observed and predicted rates of PCa BCR-free survival (**Figure 6B**). Univariable and multivariable Cox analysis further confirmed the nomogram’s ability to predict BCR in PCa patients (**Figures 6E, F**).

### 3.4 Signalling pathway analysis

**Figure 7:**
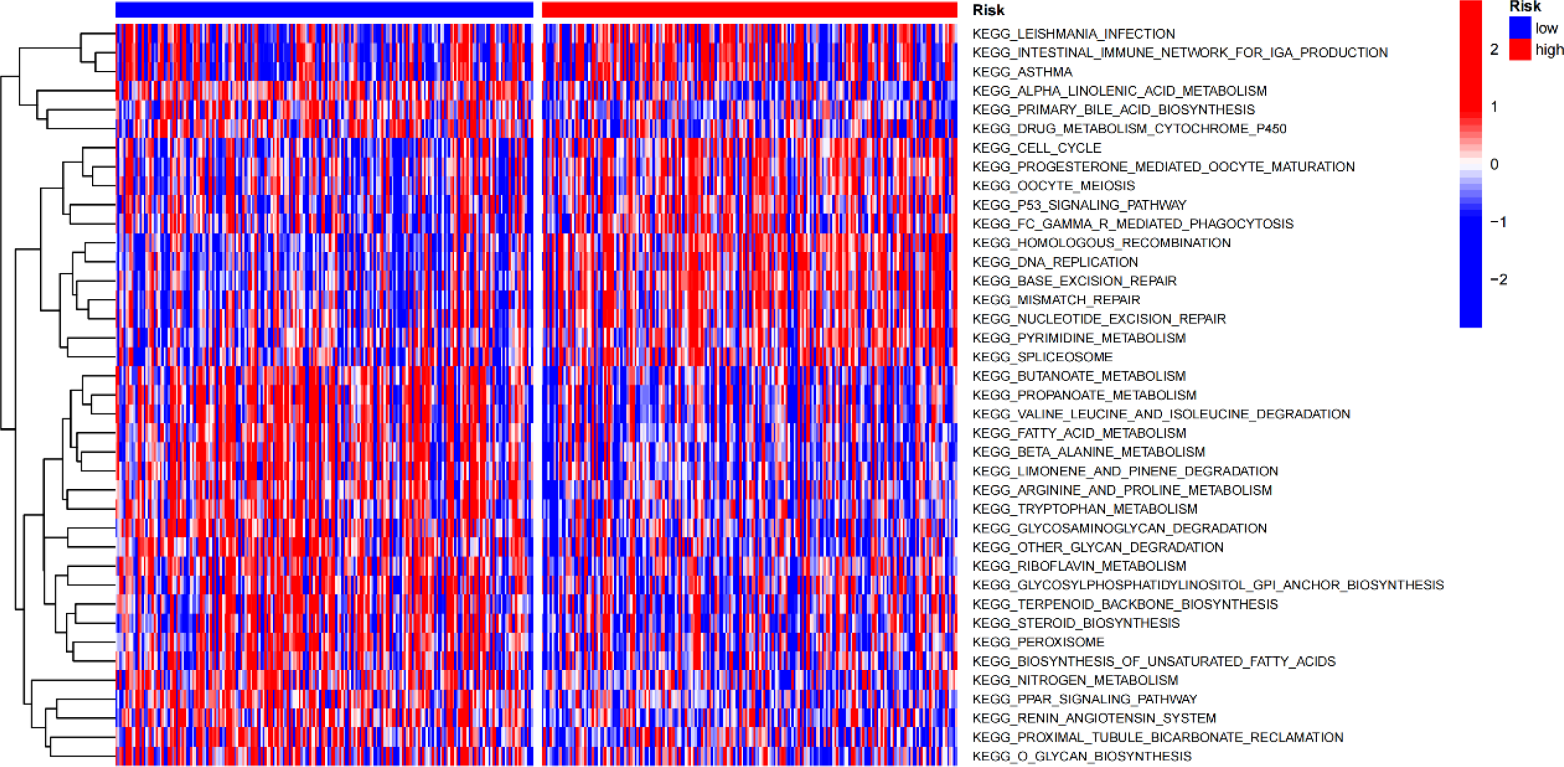
Gene set variation analysis.

**Figure 8:**
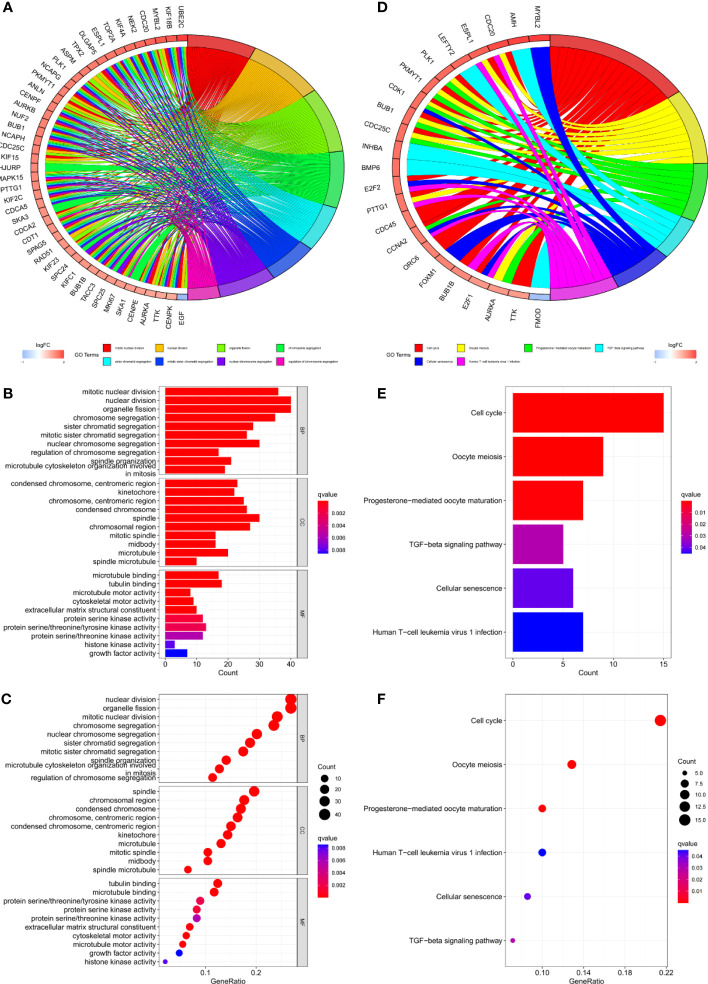
**(A–C)** GO and **(D–F)** KEGG analyses for differentially expressed genes among high and low risk groups.

Gene set variation analysis (GSVA) was used to explore potential differences in biological function and signalling pathways between the different risk groups (**Figure 7**). Results showed that many gene replication-related pathways were active in the high-risk group, including DNA replication and NER pathways. The differential genes were highly enriched in functions related to cell division, including mitotic nuclear division, nuclear division, cell cycle, etc., according to both Gene Ontology (GO) and Kyoto Encyclopedia of Genes and Genomes (KEGG) analysis (**Figure 8**).

### 3.5 Network Core Gene and Single Gene Analysis (SGA)

**Figure 9:**
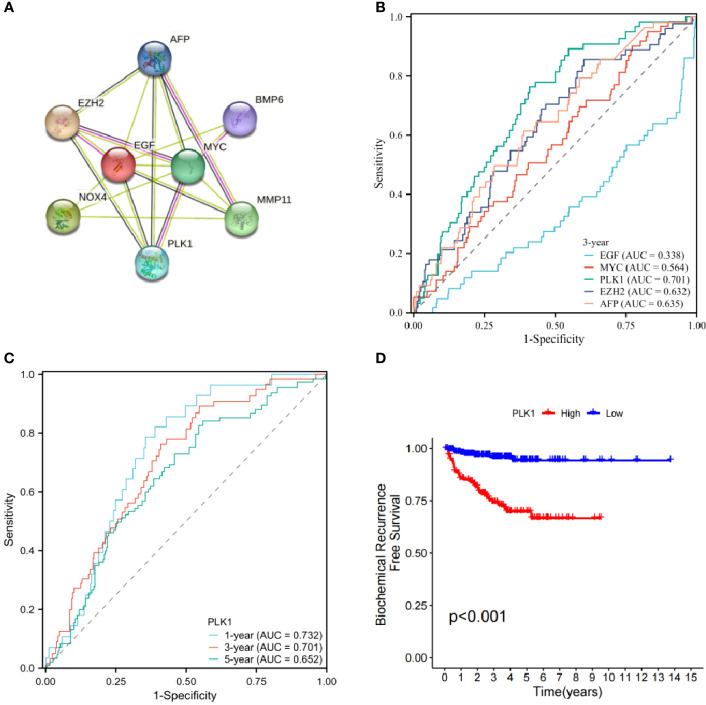
**(A)** Protein interaction network of signature genes. **(B)** ROC curve of candidate core genes. **(C)** Time-dependent ROC analysis curve for PLK1. **(D)** Single gene BCR-free survival analysis.

Protein interaction of differentially expressed genes was analysed using STRING database. Five genes (EGF, MYC, PLK1, EZH2, AFP) were identified as candidate core genes by protein interaction network (**Figure 9A**).The ROC curve showed that PLK1 was most predictive of BCR risk at 3 years post-operatively (**Figure 9B**). The areas under the AUC curve for 1, 3 and 5 year BCR-free survival were 0.732, 0.701 and 0.652, respectively, which were greater than 0.65 (**Figure 9C**). BCR-free survival analysis showed a progressive decrease in BCR-free survival over time, with marked differences in BCR-free survival between high- and low-expressing patients (**Figure 9D**).

### 3.6 Analysis of clinical correlation

**Figure 10:**
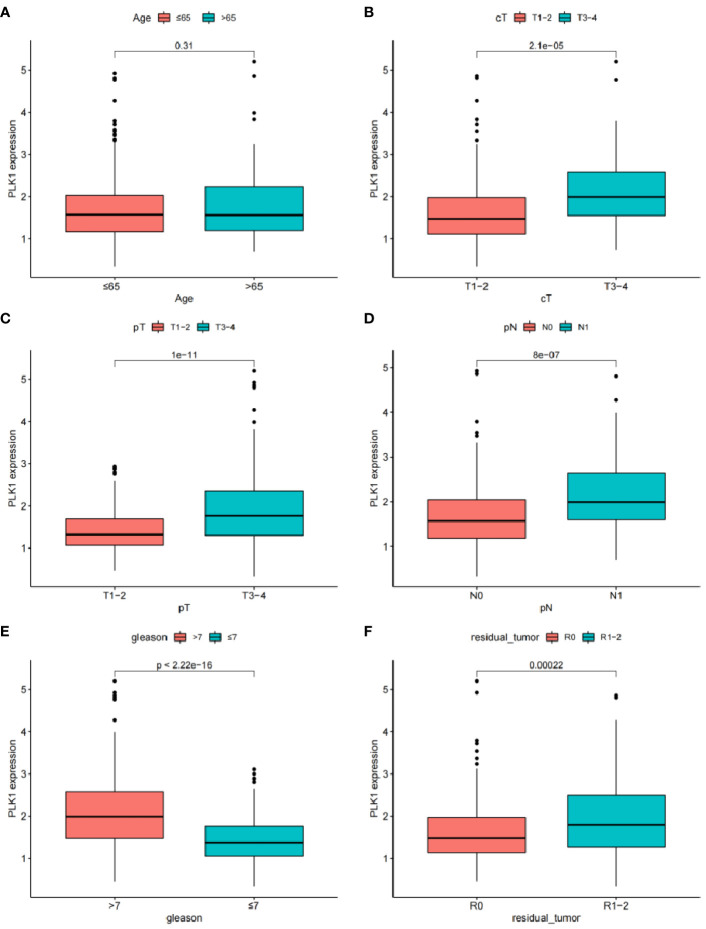
The relationship between the expression of EGF and different clinical features: **(A)** Age. **(B)** Clinical tumor. **(C)** Pathology tumor **(D)** Pathology node. **(E)** Gleason. **(F)** Residual tumor.

Clinical correlation analysis showed that PLK-1 expression level is not related to age, but is closely associated with other clinical and pathologic phenotypes (**Figures 10A–F**). PLK-1 expression can predict biochemical recurrence-free survival of PCa patients.

## 4 Discussion

With the improvement in the life expectancy of human beings and the worsening of the ageing process, the incidence rate of PCa will continue to increase. Although the treatment of PCa has evolved into a long-term comprehensive treatment modality, including active surveillance, surgery, androgen ablation, radiation therapy, and chemotherapy, it is still difficult to avoid the occurrence of BCR with the advancement of treatment modalities (15). More than half of the patients with PCa will be subjected to BCR after radical treatment, and about 35% of them will be subjected to BCR within 10 years after surgery (16). BCR is the central node of PCa progression, increasing the risk of castrate-resistant disease progression and distant metastases (17). Due to patients’ poor compliance, low follow-up frequency and insufficient follow-up time, BCR cannot be detected early enough for timely intervention. As a result, local recurrence or distant metastasis and progression to metastatic castration-resistant PCa (mCRPC) occur, and the opportunity for treatment is lost. Although medical advances and developments have significantly improved the cure rate of PCa, the malignant stage of the disease is unknown, frequently leading to non-standardised subsequent treatment.

In the long-term follow-up and monitoring of PCa patients, recurrence is the focus of attention. It is expected that PCa-related deaths can be effectively prevented by routine monitoring for BCR (18). Research findings show that the new adjuvant ADT combination regimen can provide long-term (>4 years) BCR-free benefits in high-risk patients (19). Therefore, BCR risk stratification for PCa patients is highly advisable, which may lead to more frequent surveillance, early intervention and even adjuvant treatment decisions. More and more clinicians are paying attention to precise intervention in tumour diseases with the development of precision medicine. By comprehensively analysing tumour recurrence, metastasis, progression and other related risk factors, the task of precise intervention is to determine the risk stratification of patients (3). More active follow-up and intervention measures should be taken for patients at intermediate and high risk to delay the progression of the disease, and efforts should be made to minimise unnecessary treatment for patients at low risk to reduce the burden of disease on patients. However, as PCa continues to be studied, routine indicators such as PSA detection, Gleason score and pathological stage are difficult to adapt to the needs of individual differences and complex diseases, and fail to provide accurate prediction of BCR risk in PCa (20). Further investigation of potential BCR regulatory mechanisms and development of novel clinical predictive signatures are urgently needed.

Genomic and transcriptomic analysis of tumors have shown that genetic alterations and/or differential expression can partially reveal the potential mechanism of tumour initiation and development, providing a potential target for targeted treatment of PCa (21). Intervening at the gene level will undoubtedly become an important part of treating cancer in the future. Therefore, continuing to search for target genes that may be related to the BCR of PCa is far-reaching in developing gene therapy. ARGs have attracted increasing attention from clinical and scientific researchers as a new hotspot gene. It refers to the process of programmed cell apoptosis that occurs after cells are detached from the primary extracellular matrix (22). This process is essential for the maintenance of tissue homeostasis and can remove dislocated or sloughed off cells under physiological or pathological conditions (23). The development of anoikis resistance allows tumour cells to escape anoikis when they detach from the extracellular matrix or adhere to other tissues and organs. This enables distant metastasis (24). The study suggests that while apoptosis, necrosis and autophagy are associated with tumour growth, the relationship between anoikis and tumour metastasis is more significant (25). Further experiments have shown that the endothelial cells that have acquired anoikis resistance tend to have a high rate of proliferation, a high rate of invasion and a low rate of apoptosis (13). In addition, anoikis has also been found to be closely related to the spread and high recurrence rate of glioma (26). Thus, the development of anoikis resistance is considered to be a sign that tumour cells are invading, metastasizing and relapsing.

ARGs play important roles in the progression of several tumors, especially in the process of metastasis: FAIM2 overexpression is associated with adverse clinical outcome in lung cancer (27), L1CAM affects the prognosis of endometrial cancer (EC) (28). Chen has been successful in constructing a prognostic signature using ARGs, which has shown a good predictive effect on the prognosis of EC (27). More and more studies have shown that ARGs also play a key role in the occurrence and development of PCa. ARGs, which are key to the survival of circulating tumour cells in PCa, may be related to epithelial-mesenchymal transformation in the process of PCa metastasis (25). Research has shown that the Rad9 protein is able to promote cell migration and resistance to anoikis, thereby promoting the progression of PCa (29). The nuclear localisation of parathyroid hormone-related peptide confers anoikis resistance on PCa cells, which is conducive to the metastatic progression of PCa (30). Depleting mitochondrial DNA in prostate epithelial cells induces the cell to develop anoikis resistance and enhances its invasive ability by activating the PI3K/Akt2 signalling pathway (31). Although previous studies have shown that ARGs are closely related to PCa migration and invasion activity, there has been no study on the use of ARG expression levels for BCR risk assessment in PCa patients. Based on this, we constructed a signature that can effectively predict the risk of BCR in PCa patients using ARGs, which has good predictive power for BCR risk (AUC=0.823), higher than other clinicopathological data such as PSA and Gleason scores (10, 11), and more accurate than other signatures (32, 33). A number of researchers have explored the feasibility of genetic prediction of biochemical recurrence in prostate cancer patients. Mei successfully constructed a signature based on N7-methylguanine-related genes for predicting biochemical recurrence of prostate cancer, but with a maximum AUC of 0.768 (34). An ferroptosis-related gene signature was developed to predict biochemical recurrence of prostate cancer, with a maximum predictive AUC of 0.766 (35). The lipid metabolism gene signature has also been attempted to predict biochemical recurrence in prostate cancer patients, but its predictive validity for patients from the TCGA database was not high, with an AUC of 0.734 (33).The DNA repair gene signature can also be used to predict biochemical recurrence of prostate cancer, and although it has good predictive power for biochemical recurrence within one year (AUC=0.827), its long-term predictive power is unstable (AUC[5]=0.691) (32). In this study, a model of apoptosis-associated genes was constructed and its predictive validity for biochemical recurrence in prostate cancer patients was higher and more stable. The AUCs were 0.756, 0.823 and 0.797 for 1-, 3- and 5-years, respectively, and we further constructed a predictive Norman plot with AUCs of 0.782, 0.814 and 0.830 for 1-, 3- and 5-years, respectively.

In this study, a predictive signature consisting of eight ARGs [EGF (36), MYC (37), PLK1 (38), EZH2 (39), AFP (40), NOX4 (41), BMP6 (42), MMP11 (43)] was established and its correlation with anoikis was verified by a literature search. EGF is not only involved in regulating the physiological function of the prostate, but also induces PCa cell proliferation and invasion (44).The polymorphism of EGF may lead to early recurrence in PCa patients treated with androgen blockade, which is expected to become a potential therapeutic target for castration-resistant PCa (45). MYC is a proto-oncogene, and its activatio1n is the basis for the initiation and development of PCa (46). The gene copy number and the expression level of MYC are closely related to the severity of PCa: MYC is significantly over-expressed during the progression of PCa, and the amplification of MYC may be associated with a higher Gleason score (47). However, even in the stage of prostate intraepithelial neoplasia, an increase in the number of copies of the MYC gene can be observed (48). Further studies are therefore needed to investigate the role of MYC in PCa. In the onset and development of PCa, PLK1 also plays an important role. Overexpressed PLK1 in Pten-deficient male mice can induce PCa, and the high expression of PLK1 is positively correlated with the high level of PCa (49). Furthermore, PLK1 is involved with the biological process of regulating other gene products, such as the activation of MYC, suggesting a possible regulatory node for the onset and development of PCa. EZH2, an epigenetic regulator, overexpressed in various tumours, can help cells acquire invasive properties (50). Studies have shown that overexpression of the EZH2 enhancer in PCa leads to negative regulation of the interferon-stimulated gene, thereby reducing the therapeutic effect of tumour immune checkpoint blockade in PCa (51).EZH2 plays its oncogenic role by acting as a co-activator of a number of key transcription factors in metastatic castration-resistant PCa cells (52). AFP, an important serological marker for hepatocellular carcinoma, teratoma and other cancers, is mainly expressed in the liver and the yolk sac of the foetus (53). As a peptide derived from the active site of AFP, the AFP peptide is a potential growth factor for PCa. AFP peptide showed a specific effect on the PCa cell line DU-145. It significantly increased its proliferative activity (54). A group of gene markers including AFP showed good predictive ability for the risk of postoperative BCR in PCa patients with Gleason score≥7 (53).NOX4 is widely distributed in the matrix of PCa and has been shown to be involved in transforming primary prostate fibroblasts into cancer-related fibroblasts (55). Elevated NOX4 expression was observed in prostate patients with reduced survival. Down-regulation of NOX4 expression provides theoretical support for targeted matrix therapy of PCa, as it is expected to inhibit the production process of PCa cells and increase the rate of apoptosis (56). In comparison with benign prostate hyperplasia, the expression of BMP-6 is upregulated in PCa, especially in metastatic PCa. BMP-6 has been shown to significantly increase the migration and invasion ability of PCa cells in *in vitro* cell experiments. It promotes PCa metastasis, particularly the process of bone metastasis, which is an indicator of poor clinical outcome (57). MMP11 expression is thought to be associated with clinical outcome in various tumours (58). Higher levels of MMP11 have been observed in patients with advanced, high-grade, metastatic and castration-resistant PCa, which is often associated with a shorter survival time (58, 59). In our study, MMP-11, which promotes BCR in PCa, is also associated with poor patient prognosis.

We further analyzed the protein interactions of 8 model genes in the STRING database and obtained the protein interaction network (**Figure 9A**). The results showed that EGF, MYC, PLK1, EZH2, and AFP have significant interactions with each other and with other genes, indicating potential as candidate network core genes. In order to further evaluate the predictive potential of the aforementioned genes for the risk of BCR in patients, we plotted ROC curves, which showed that among the core genes in the candidate network, the expression level of PLK1 was the most accurate in predicting the risk of BCR in patients. The areas under ROC curve of 1-, 3- and 5 years were respectively 0.732, 0.701 and 0.652.Therefore, we ultimately chose PLK1 as the network core gene for our study.

PLK1 is a cell cycle-regulating serine/threonine kinase that plays a critical role in the dominant division process of normal and cancerous cells (60). It has been confirmed that PLK1 is overexpressed in various human malignant tumour tissues, and PLK1 expression level significantly correlates positively with tumour cell proliferation ability, metastatic potential and poor prognosis (61). Cell separation can induce upregulation of PLK1 expression, and high levels of PLK1 enhance cancer cell resistance to anoikis through regulation of b-catenin expression (38). Depleting PLK1 can significantly inhibit tumour cell proliferation *in vitro* and induce tumour cell apoptosis (62). Researchers have become interested in studying urothelial tumours because of the regulatory role of PLK1 in a variety of tumours. Immunohistochemistry has shown that PLK1 is also significantly overexpressed in PCa tissue, and clinical correlation analysis showed a positive correlation between its expression level and Gleason score (62). *In vitro* cell experiments have confirmed that upregulation of PLK1 expression can induce epithelial-mesenchymal transformation of prostate epithelial cells, increasing their migration ability and malignant transformation potential (61); animal experiments have found a significant increase in PLK1 expression in PCa xenograft animal models with castration resistance, suggesting that PLK1 may promote disease progression in PCa (63). PLK1 may be a key factor in initiating and developing PCa, but unfortunately researchers have failed to correlate PLK1 expression levels with patient prognosis. Therefore, by performing a BCR-free survival analysis, we investigated the feasibility of using PLK1 alone to predict BCR in patients. The survival curve showed a significant difference in BCR-free survival between the high and low PLK1 expression groups; clinical correlation analysis showed that PLK1 expression level was not related to age, but was significantly associated with clinicopathological characteristics such as T stage. Our study not only provides further confirmation of the promoting effect of PLK1 on the malignant phenotype of PCa, but also suggests that there is a correlation between PLK1 and its BCR in PCa patients. It is possible that PLK1 may have an impact on the prognosis of PCa patients through a variety of pathways. Targeting PLK1 may be an effective addition to the diagnosis and treatment of PCa through further research into its underlying mechanisms.

There are several limitations to this study. Firstly, the retrospective studies that have been carried out must be validated by numerous prospective experiments. Secondly, although we have demonstrated a difference in prognosis between the high-risk group and the low-risk group, it remains to be investigated whether there is a difference in the response to adjuvant therapy, such as immunotherapy, between the two groups. Finally, the specific mechanism of ARGs’ effect on PCa BCR is still unknown. More work needs to be done to elucidate the mechanism of action and to identify targets for clinical diagnosis and therapy.

## 5 Conclusions

To summarise, we constructed a characteristic consisting of 8 ARGs. It is possible to classify PCa patients into a high-risk, and a low-risk, group. The clinicopathological characteristics and the risk of BCR are significantly different between the two groups. Stricter clinical surveillance and even adjuvant therapy may be required in high-risk patients. As a potential core gene, PLK1 may play a key regulatory role. This study is a new and effective tool for risk assessment of BCR in PCa and confirms the clinical value of in-depth investigation of ARGs in PCa.

## Data availability statement

Publicly available datasets were analyzed in this study. This data can be found here: TCGA, https://portal.gdc.cancer.gov/, GEO, https://www.ncbi.nlm.nih.gov/, HPA, https://www.proteinatlas.org/, GeneCard, https://www.genecards.org/.

## Ethics statement

Written informed consent was obtained from the individual(s) for the publication of any potentially identifiable images or data included in this article.

## Author contributions

XZ is responsible for research design and manuscript writing, ZW and ZT are jointly responsible for statistical analysis and graphics drawing. JH and YZ are responsible for manuscript and graphic modification. JG, JD and SX provided academic and scientific guidance. All authors have reviewed and approved the manuscript.
