# Supplementary material for: An anoikis-related gene signature for prediction of the prognosis in prostate cancer
Source: Front Oncol. 2023 Aug 17;13:1169425. doi: 10.3389/fonc.2023.1169425 (PMC10469923; doi:10.3389/fonc.2023.1169425)
Supplement: Supplementary file 1 [file Table_1.docx]

Supplementary Table 1: The list of 338 anoikis-related genes (Relevance score≥1).

| Gene Symbol | Description | Relevance score |
| --- | --- | --- |
| BRMS1 | BRMS1 Transcriptional Repressor And Anoikis Regulator | 14.70990 |
| PTK2 | Protein Tyrosine Kinase 2 | 7.29451 |
| NTRK2 | Neurotrophic Receptor Tyrosine Kinase 2 | 7.26745 |
| BCL2L11 | BCL2 Like 11 | 6.69656 |
| SRC | SRC Proto-Oncogene, Non-Receptor Tyrosine Kinase | 6.19012 |
| CEACAM6 | CEA Cell Adhesion Molecule 6 | 6.10605 |
| CAV1 | Caveolin 1 | 5.46839 |
| AKT1 | AKT Serine/Threonine Kinase 1 | 5.41247 |
| ITGB1 | Integrin Subunit Beta 1 | 5.00751 |
| CEACAM5 | CEA Cell Adhesion Molecule 5 | 4.65795 |
| EGFR | Epidermal Growth Factor Receptor | 4.62049 |
| BCL2 | BCL2 Apoptosis Regulator | 4.54555 |
| CASP8 | Caspase 8 | 4.49354 |
| SIK1 | Salt Inducible Kinase 1 | 4.37666 |
| PTRH2 | Peptidyl-TRNA Hydrolase 2 | 4.19639 |
| STAT3 | Signal Transducer And Activator Of Transcription 3 | 4.14507 |
| TLE1 | TLE Family Member 1, Transcriptional Corepressor | 4.07276 |
| DAPK2 | Death Associated Protein Kinase 2 | 3.98992 |
| CTNNB1 | Catenin Beta 1 | 3.97906 |
| ZNF304 | Zinc Finger Protein 304 | 3.94151 |
| MAPK1 | Mitogen-Activated Protein Kinase 1 | 3.73843 |
| BMF | Bcl2 Modifying Factor | 3.73578 |
| ITGA5 | Integrin Subunit Alpha 5 | 3.68077 |
| TP53 | Tumor Protein P53 | 3.64358 |
| MCL1 | MCL1 Apoptosis Regulator, BCL2 Family Member | 3.58216 |
| BCL2L1 | BCL2 Like 1 | 3.36490 |
| CASP3 | Caspase 3 | 3.12323 |
| CDH1 | Cadherin 1 | 3.06282 |
| BAD | BCL2 Associated Agonist Of Cell Death | 2.96617 |
| PIK3CA | Phosphatidylinositol-4,5-Bisphosphate 3-Kinase Catalytic Subunit Alpha | 2.94418 |
| PAK1 | P21 (RAC1) Activated Kinase 1 | 2.93396 |
| ITGAV | Integrin Subunit Alpha V | 2.87833 |
| FN1 | Fibronectin 1 | 2.82443 |
| MAPK3 | Mitogen-Activated Protein Kinase 3 | 2.73272 |
| PTGS2 | Prostaglandin-Endoperoxide Synthase 2 | 2.69496 |
| BAX | BCL2 Associated X, Apoptosis Regulator | 2.55082 |
| BCAR1 | BCAR1 Scaffold Protein, Cas Family Member | 2.55082 |
| PTEN | Phosphatase And Tensin Homolog | 2.52112 |
| ERBB2 | Erb-B2 Receptor Tyrosine Kinase 2 | 2.43650 |
| PDK4 | Pyruvate Dehydrogenase Kinase 4 | 2.41514 |
| ANGPTL4 | Angiopoietin Like 4 | 2.40092 |
| CYCS | Cytochrome C, Somatic | 2.34194 |
| BRAF | B-Raf Proto-Oncogene, Serine/Threonine Kinase | 2.33776 |
| YAP1 | Yes1 Associated Transcriptional Regulator | 2.33462 |
| ANKRD13C | Ankyrin Repeat Domain 13C | 2.32947 |
| ITGA2 | Integrin Subunit Alpha 2 | 2.30060 |
| ANXA5 | Annexin A5 | 2.26835 |
| BIRC5 | Baculoviral IAP Repeat Containing 5 | 2.25808 |
| MTOR | Mechanistic Target Of Rapamycin Kinase | 2.25142 |
| TIMP1 | TIMP Metallopeptidase Inhibitor 1 | 2.24774 |
| BDNF | Brain Derived Neurotrophic Factor | 2.22380 |
| CSPG4 | Chondroitin Sulfate Proteoglycan 4 | 2.19748 |
| BSG | Basigin (Ok Blood Group) | 2.19748 |
| AKT2 | AKT Serine/Threonine Kinase 2 | 2.18487 |
| STK11 | Serine/Threonine Kinase 11 | 2.15194 |
| IGF1 | Insulin Like Growth Factor 1 | 2.15000 |
| IGF1R | Insulin Like Growth Factor 1 Receptor | 2.14920 |
| ITGA6 | Integrin Subunit Alpha 6 | 2.11204 |
| ILK | Integrin Linked Kinase | 2.08539 |
| CFLAR | CASP8 And FADD Like Apoptosis Regulator | 2.08519 |
| RHOA | Ras Homolog Family Member A | 2.07076 |
| HIF1A | Hypoxia Inducible Factor 1 Subunit Alpha | 2.06712 |
| DAP3 | Death Associated Protein 3 | 2.05750 |
| MYBBP1A | MYB Binding Protein 1a | 2.02231 |
| ITGA3 | Integrin Subunit Alpha 3 | 2.00020 |
| TLE5 | TLE Family Member 5, Transcriptional Modulator | 1.99963 |
| PTK2B | Protein Tyrosine Kinase 2 Beta | 1.99717 |
| CCND1 | Cyclin D1 | 1.98345 |
| CTTN | Cortactin | 1.98345 |
| CALR | Calreticulin | 1.94678 |
| ATF4 | Activating Transcription Factor 4 | 1.94678 |
| CDCP1 | CUB Domain Containing Protein 1 | 1.93394 |
| SKP2 | S-Phase Kinase Associated Protein 2 | 1.90986 |
| CHEK2 | Checkpoint Kinase 2 | 1.90856 |
| HGF | Hepatocyte Growth Factor | 1.88534 |
| E2F1 | E2F Transcription Factor 1 | 1.88129 |
| EGF | Epidermal Growth Factor | 1.87265 |
| PIK3CG | Phosphatidylinositol-4,5-Bisphosphate 3-Kinase Catalytic Subunit Gamma | 1.86941 |
| ITGB4 | Integrin Subunit Beta 4 | 1.85761 |
| DAPK1 | Death Associated Protein Kinase 1 | 1.84749 |
| PIK3R1 | Phosphoinositide-3-Kinase Regulatory Subunit 1 | 1.82258 |
| PIK3R3 | Phosphoinositide-3-Kinase Regulatory Subunit 3 | 1.81689 |
| MAP2K1 | Mitogen-Activated Protein Kinase Kinase 1 | 1.79803 |
| CXCL12 | C-X-C Motif Chemokine Ligand 12 | 1.77875 |
| LGALS3 | Galectin 3 | 1.74328 |
| FBXW7-AS1 | FBXW7 Antisense RNA 1 | 1.73565 |
| BAK1 | BCL2 Antagonist/Killer 1 | 1.73191 |
| ABHD4 | Abhydrolase Domain Containing 4, N-Acyl Phospholipase B | 1.70824 |
| CD44 | CD44 Molecule (Indian Blood Group) | 1.70472 |
| ITGA4 | Integrin Subunit Alpha 4 | 1.69146 |
| FADD | Fas Associated Via Death Domain | 1.69146 |
| PHLDA2 | Pleckstrin Homology Like Domain Family A Member 2 | 1.69146 |
| TGFB1 | Transforming Growth Factor Beta 1 | 1.68723 |
| HMCN1 | Hemicentin 1 | 1.68723 |
| MMP2 | Matrix Metallopeptidase 2 | 1.67407 |
| CEBPB | CCAAT Enhancer Binding Protein Beta | 1.67407 |
| CEMIP | Cell Migration Inducing Hyaluronidase 1 | 1.67407 |
| CDKN3 | Cyclin Dependent Kinase Inhibitor 3 | 1.66837 |
| CBL | Cbl Proto-Oncogene | 1.65600 |
| CASP9 | Caspase 9 | 1.65600 |
| SFN | Stratifin | 1.65600 |
| MTDH | Metadherin | 1.65600 |
| PRKCA | Protein Kinase C Alpha | 1.63716 |
| TNFRSF10B | TNF Receptor Superfamily Member 10b | 1.63716 |
| CXCL8 | C-X-C Motif Chemokine Ligand 8 | 1.63716 |
| MIR200C | MicroRNA 200c | 1.63716 |
| AR | Androgen Receptor | 1.62012 |
| CDKN2A | Cyclin Dependent Kinase Inhibitor 2A | 1.61744 |
| MAPK8 | Mitogen-Activated Protein Kinase 8 | 1.61744 |
| CPT1A | Carnitine Palmitoyltransferase 1A | 1.61744 |
| PIK3CB | Phosphatidylinositol-4,5-Bisphosphate 3-Kinase Catalytic Subunit Beta | 1.61744 |
| CLDN1 | Claudin 1 | 1.61744 |
| MIR204 | MicroRNA 204 | 1.61744 |
| MIR26A1 | MicroRNA 26a-1 | 1.61744 |
| CDKN1A | Cyclin Dependent Kinase Inhibitor 1A | 1.59670 |
| CDKN1B | Cyclin Dependent Kinase Inhibitor 1B | 1.59670 |
| KLF12 | Kruppel Like Factor 12 | 1.59670 |
| NTRK1 | Neurotrophic Receptor Tyrosine Kinase 1 | 1.57522 |
| PLAU | Plasminogen Activator, Urokinase | 1.57478 |
| MYC | MYC Proto-Oncogene, BHLH Transcription Factor | 1.57478 |
| PLK1 | Polo Like Kinase 1 | 1.57478 |
| SMAD4 | SMAD Family Member 4 | 1.57478 |
| MUC1 | Mucin 1, Cell Surface Associated | 1.57478 |
| PLAUR | Plasminogen Activator, Urokinase Receptor | 1.57478 |
| LGALS1 | Galectin 1 | 1.57478 |
| PYCARD | PYD And CARD Domain Containing | 1.57478 |
| SESN2 | Sestrin 2 | 1.57478 |
| ITGB3 | Integrin Subunit Beta 3 | 1.57011 |
| KRAS | KRAS Proto-Oncogene, GTPase | 1.57011 |
| THBS1 | Thrombospondin 1 | 1.55144 |
| BID | BH3 Interacting Domain Death Agonist | 1.55144 |
| HRAS | HRas Proto-Oncogene, GTPase | 1.53666 |
| CDK11B | Cyclin Dependent Kinase 11B | 1.52636 |
| CDK11A | Cyclin Dependent Kinase 11A | 1.52636 |
| XIAP | X-Linked Inhibitor Of Apoptosis | 1.51971 |
| PPARG | Peroxisome Proliferator Activated Receptor Gamma | 1.49910 |
| IL6 | Interleukin 6 | 1.49910 |
| MIR145 | MicroRNA 145 | 1.49910 |
| CCR7 | C-C Motif Chemokine Receptor 7 | 1.46893 |
| MSLN | Mesothelin | 1.46893 |
| RAC1 | Rac Family Small GTPase 1 | 1.46308 |
| GRHL2 | Grainyhead Like Transcription Factor 2 | 1.46308 |
| NOTCH1 | Notch Receptor 1 | 1.43893 |
| RHOG | Ras Homolog Family Member G | 1.43769 |
| CCAR2 | Cell Cycle And Apoptosis Regulator 2 | 1.43769 |
| NQO1 | NAD(P)H Quinone Dehydrogenase 1 | 1.43470 |
| BIRC3 | Baculoviral IAP Repeat Containing 3 | 1.43243 |
| MMP13 | Matrix Metallopeptidase 13 | 1.40008 |
| FAS | Fas Cell Surface Death Receptor | 1.39708 |
| MTA1 | Metastasis Associated 1 | 1.39708 |
| MYO5A | Myosin VA | 1.39409 |
| EDA2R | Ectodysplasin A2 Receptor | 1.39409 |
| CCN6 | Cellular Communication Network Factor 6 | 1.39409 |
| MMP9 | Matrix Metallopeptidase 9 | 1.37579 |
| ABL1 | ABL Proto-Oncogene 1, Non-Receptor Tyrosine Kinase | 1.37579 |
| MAPK11 | Mitogen-Activated Protein Kinase 11 | 1.37579 |
| PTHLH | Parathyroid Hormone Like Hormone | 1.37200 |
| PDGFB | Platelet Derived Growth Factor Subunit B | 1.35506 |
| GLI2 | GLI Family Zinc Finger 2 | 1.35506 |
| EZH2 | Enhancer Of Zeste 2 Polycomb Repressive Complex 2 Subunit | 1.35392 |
| CXCR4 | C-X-C Motif Chemokine Receptor 4 | 1.34474 |
| RIPK1 | Receptor Interacting Serine/Threonine Kinase 1 | 1.33607 |
| HMGA1 | High Mobility Group AT-Hook 1 | 1.33314 |
| SIK2 | Salt Inducible Kinase 2 | 1.33314 |
| TNFSF10 | TNF Superfamily Member 10 | 1.33314 |
| ANGPTL2 | Angiopoietin Like 2 | 1.31457 |
| S100A4 | S100 Calcium Binding Protein A4 | 1.30980 |
| ETV4 | ETS Variant Transcription Factor 4 | 1.30980 |
| NTF3 | Neurotrophin 3 | 1.30980 |
| MIR21 | MicroRNA 21 | 1.30980 |
| MIR124-1 | MicroRNA 124-1 | 1.30980 |
| HTRA1 | HtrA Serine Peptidase 1 | 1.28472 |
| LATS1 | Large Tumor Suppressor Kinase 1 | 1.28472 |
| CEACAM3 | CEA Cell Adhesion Molecule 3 | 1.28472 |
| EIF2AK3 | Eukaryotic Translation Initiation Factor 2 Alpha Kinase 3 | 1.28176 |
| LAMC2 | Laminin Subunit Gamma 2 | 1.28176 |
| LAMA3 | Laminin Subunit Alpha 3 | 1.28176 |
| LAMB3 | Laminin Subunit Beta 3 | 1.28176 |
| CDH2 | Cadherin 2 | 1.26204 |
| CSNK2A1 | Casein Kinase 2 Alpha 1 | 1.26204 |
| EDIL3 | EGF Like Repeats And Discoidin Domains 3 | 1.26204 |
| ZEB2 | Zinc Finger E-Box Binding Homeobox 2 | 1.25745 |
| TLN1 | Talin 1 | 1.25745 |
| EPHA2 | EPH Receptor A2 | 1.24131 |
| SOD2 | Superoxide Dismutase 2 | 1.24131 |
| SIRT3 | Sirtuin 3 | 1.24131 |
| OLFM3 | Olfactomedin 3 | 1.24131 |
| CLU | Clusterin | 1.22729 |
| SPINK1 | Serine Peptidase Inhibitor Kazal Type 1 | 1.22729 |
| CPEB2 | Cytoplasmic Polyadenylation Element Binding Protein 2 | 1.22729 |
| NAT1 | N-Acetyltransferase 1 | 1.21938 |
| TSG101 | Tumor Susceptibility 101 | 1.21938 |
| MIR200A | MicroRNA 200a | 1.21938 |
| MIR6744 | MicroRNA 6744 | 1.21938 |
| SERPINA1 | Serpin Family A Member 1 | 1.21340 |
| AKT3 | AKT Serine/Threonine Kinase 3 | 1.19604 |
| RELA | RELA Proto-Oncogene, NF-KB Subunit | 1.19604 |
| TNFRSF1A | TNF Receptor Superfamily Member 1A | 1.19604 |
| AFP | Alpha Fetoprotein | 1.19604 |
| FASLG | Fas Ligand | 1.19604 |
| EEF1A1 | Eukaryotic Translation Elongation Factor 1 Alpha 1 | 1.19604 |
| ITGA8 | Integrin Subunit Alpha 8 | 1.19604 |
| SATB1 | SATB Homeobox 1 | 1.19604 |
| CD63 | CD63 Molecule | 1.19604 |
| LTB4R2 | Leukotriene B4 Receptor 2 | 1.19604 |
| NOX4 | NADPH Oxidase 4 | 1.19604 |
| PBK | PDZ Binding Kinase | 1.19604 |
| MAVS | Mitochondrial Antiviral Signaling Protein | 1.19604 |
| HRC | Histidine Rich Calcium Binding Protein | 1.19604 |
| RHOB | Ras Homolog Family Member B | 1.19305 |
| CCN2 | Cellular Communication Network Factor 2 | 1.19305 |
| PPP1R13B | Protein Phosphatase 1 Regulatory Subunit 13B | 1.19305 |
| PLG | Plasminogen | 1.18681 |
| MET | MET Proto-Oncogene, Receptor Tyrosine Kinase | 1.18547 |
| RAF1 | Raf-1 Proto-Oncogene, Serine/Threonine Kinase | 1.17097 |
| PARP1 | Poly(ADP-Ribose) Polymerase 1 | 1.17097 |
| PRKCQ | Protein Kinase C Theta | 1.17097 |
| BRCA2 | BRCA2 DNA Repair Associated | 1.17097 |
| RB1 | RB Transcriptional Corepressor 1 | 1.17097 |
| DOCK1 | Dedicator Of Cytokinesis 1 | 1.17097 |
| HAVCR2 | Hepatitis A Virus Cellular Receptor 2 | 1.17097 |
| SP1 | Sp1 Transcription Factor | 1.17097 |
| VTN | Vitronectin | 1.17097 |
| INHBB | Inhibin Subunit Beta B | 1.17097 |
| PDCD4 | Programmed Cell Death 4 | 1.17097 |
| PRPF4B | Pre-MRNA Processing Factor 4B | 1.17097 |
| RANBP9 | RAN Binding Protein 9 | 1.17097 |
| SESN1 | Sestrin 1 | 1.17097 |
| SESN3 | Sestrin 3 | 1.17097 |
| ZBTB7A | Zinc Finger And BTB Domain Containing 7A | 1.17097 |
| CD24 | CD24 Molecule | 1.17097 |
| MIR141 | MicroRNA 141 | 1.17097 |
| ELANE | Elastase, Neutrophil Expressed | 1.15244 |
| KDR | Kinase Insert Domain Receptor | 1.14370 |
| MDM2 | MDM2 Proto-Oncogene | 1.14370 |
| NFE2L2 | NFE2 Like BZIP Transcription Factor 2 | 1.14370 |
| PRKCI | Protein Kinase C Iota | 1.14370 |
| ZEB1 | Zinc Finger E-Box Binding Homeobox 1 | 1.14370 |
| HK2 | Hexokinase 2 | 1.14370 |
| KL | Klotho | 1.14370 |
| CRYAB | Crystallin Alpha B | 1.14370 |
| EPHB6 | EPH Receptor B6 | 1.14370 |
| FGF2 | Fibroblast Growth Factor 2 | 1.14370 |
| LTF | Lactotransferrin | 1.14370 |
| IQGAP1 | IQ Motif Containing GTPase Activating Protein 1 | 1.14370 |
| MGAT5 | Alpha-1,6-Mannosylglycoprotein 6-Beta-N-Acetylglucosaminyltransferase | 1.14370 |
| SDCBP | Syndecan Binding Protein | 1.14370 |
| ABHD2 | Abhydrolase Domain Containing 2, Acylglycerol Lipase | 1.14370 |
| SPIB | Spi-B Transcription Factor | 1.14370 |
| TRIM31 | Tripartite Motif Containing 31 | 1.14370 |
| MIR1827 | MicroRNA 1827 | 1.14370 |
| PDGFRB | Platelet Derived Growth Factor Receptor Beta | 1.11354 |
| TLR3 | Toll Like Receptor 3 | 1.11354 |
| PLAT | Plasminogen Activator, Tissue Type | 1.11354 |
| ROCK1 | Rho Associated Coiled-Coil Containing Protein Kinase 1 | 1.11354 |
| NRAS | NRAS Proto-Oncogene, GTPase | 1.11354 |
| CASP10 | Caspase 10 | 1.11354 |
| PAK4 | P21 (RAC1) Activated Kinase 4 | 1.11354 |
| VEGFA | Vascular Endothelial Growth Factor A | 1.11354 |
| PIN1 | Peptidylprolyl Cis/Trans Isomerase, NIMA-Interacting 1 | 1.11354 |
| YWHAZ | Tyrosine 3-Monooxygenase/Tryptophan 5-Monooxygenase Activation Protein Zeta | 1.11354 |
| TWIST1 | Twist Family BHLH Transcription Factor 1 | 1.11354 |
| UBE2C | Ubiquitin Conjugating Enzyme E2 C | 1.11354 |
| IL1RAP | Interleukin 1 Receptor Accessory Protein | 1.11354 |
| BMP6 | Bone Morphogenetic Protein 6 | 1.11354 |
| ELK1 | ETS Transcription Factor ELK1 | 1.11354 |
| PRDX4 | Peroxiredoxin 4 | 1.11354 |
| BNIP3 | BCL2 Interacting Protein 3 | 1.11354 |
| BNIP3L | BCL2 Interacting Protein 3 Like | 1.11354 |
| KDM3A | Lysine Demethylase 3A | 1.11354 |
| LMO3 | LIM Domain Only 3 | 1.11354 |
| ZNF32 | Zinc Finger Protein 32 | 1.11354 |
| MIR200B | MicroRNA 200b | 1.11354 |
| MIR525 | MicroRNA 525 | 1.11354 |
| MIR363 | MicroRNA 363 | 1.11354 |
| TUBB3 | Tubulin Beta 3 Class III | 1.09952 |
| HSP90B1 | Heat Shock Protein 90 Beta Family Member 1 | 1.09952 |
| PTPN11 | Protein Tyrosine Phosphatase Non-Receptor Type 11 | 1.07930 |
| SLC2A1 | Solute Carrier Family 2 Member 1 | 1.07930 |
| HMOX1 | Heme Oxygenase 1 | 1.07930 |
| PRKACA | Protein Kinase CAMP-Activated Catalytic Subunit Alpha | 1.07930 |
| PAK3 | P21 (RAC1) Activated Kinase 3 | 1.07930 |
| PIK3R2 | Phosphoinositide-3-Kinase Regulatory Subunit 2 | 1.07930 |
| PPP2CA | Protein Phosphatase 2 Catalytic Subunit Alpha | 1.07930 |
| CASP6 | Caspase 6 | 1.07930 |
| CD36 | CD36 Molecule | 1.07930 |
| CDH3 | Cadherin 3 | 1.07930 |
| LRP1 | LDL Receptor Related Protein 1 | 1.07930 |
| PTK6 | Protein Tyrosine Kinase 6 | 1.07930 |
| EEF2K | Eukaryotic Elongation Factor 2 Kinase | 1.07930 |
| GLO1 | Glyoxalase I | 1.07930 |
| LPAR1 | Lysophosphatidic Acid Receptor 1 | 1.07930 |
| PAK2 | P21 (RAC1) Activated Kinase 2 | 1.07930 |
| ADCY10 | Adenylate Cyclase 10 | 1.07930 |
| RBL2 | RB Transcriptional Corepressor Like 2 | 1.07930 |
| CEACAM1 | CEA Cell Adhesion Molecule 1 | 1.07930 |
| GDF2 | Growth Differentiation Factor 2 | 1.07930 |
| SIRPA | Signal Regulatory Protein Alpha | 1.07930 |
| TRAF2 | TNF Receptor Associated Factor 2 | 1.07930 |
| APOBEC3G | Apolipoprotein B MRNA Editing Enzyme Catalytic Subunit 3G | 1.07930 |
| MNX1 | Motor Neuron And Pancreas Homeobox 1 | 1.07930 |
| TNFRSF12A | TNF Receptor Superfamily Member 12A | 1.07930 |
| VPS37A | VPS37A Subunit Of ESCRT-I | 1.07930 |
| BAG1 | BAG Cochaperone 1 | 1.07930 |
| IL17A | Interleukin 17A | 1.07930 |
| COL13A1 | Collagen Type XIII Alpha 1 Chain | 1.07930 |
| RAD9A | RAD9 Checkpoint Clamp Component A | 1.07930 |
| IFI27 | Interferon Alpha Inducible Protein 27 | 1.07930 |
| MEGF11 | Multiple EGF Like Domains 11 | 1.07930 |
| ITPRIP | Inositol 1,4,5-Trisphosphate Receptor Interacting Protein | 1.07930 |
| BCL2L15 | BCL2 Like 15 | 1.07930 |
| SNAI2 | Snail Family Transcriptional Repressor 2 | 1.05056 |
| GLUD1 | Glutamate Dehydrogenase 1 | 1.03869 |
| NOTCH3 | Notch Receptor 3 | 1.03869 |
| PTPN1 | Protein Tyrosine Phosphatase Non-Receptor Type 1 | 1.03869 |
| FASN | Fatty Acid Synthase | 1.03869 |
| MYH9 | Myosin Heavy Chain 9 | 1.03869 |
| RPS6KB1 | Ribosomal Protein S6 Kinase B1 | 1.03869 |
| SIRT1 | Sirtuin 1 | 1.03869 |
| TPM1 | Tropomyosin 1 | 1.03869 |
| PPP2R1A | Protein Phosphatase 2 Scaffold Subunit Aalpha | 1.03869 |
| COL4A2 | Collagen Type IV Alpha 2 Chain | 1.03869 |
| CTNND1 | Catenin Delta 1 | 1.03869 |
| MMP11 | Matrix Metallopeptidase 11 | 1.03869 |
| CD151 | CD151 Molecule (Raph Blood Group) | 1.03869 |
| PPP2R2A | Protein Phosphatase 2 Regulatory Subunit Balpha | 1.03869 |
| SEMA7A | Semaphorin 7A (John Milton Hagen Blood Group) | 1.03869 |
| ARHGEF7 | Rho Guanine Nucleotide Exchange Factor 7 | 1.03869 |
| BST2 | Bone Marrow Stromal Cell Antigen 2 | 1.03869 |
| PPP2R5A | Protein Phosphatase 2 Regulatory Subunit B'Alpha | 1.03869 |
| PPP2R2D | Protein Phosphatase 2 Regulatory Subunit Bdelta | 1.03869 |
| CCN1 | Cellular Communication Network Factor 1 | 1.03869 |
| CCDC178 | Coiled-Coil Domain Containing 178 | 1.03869 |
| MIR10A | MicroRNA 10a | 1.03869 |
| MIR30C1 | MicroRNA 30c-1 | 1.03869 |
| MIR30B | MicroRNA 30b | 1.03869 |
| SHC1 | SHC Adaptor Protein 1 | 1.01773 |
